# The BRAF-inhibitor PLX4720 inhibits CXCL8 secretion in BRAFV600E mutated and normal thyroid cells: a further anti-cancer effect of BRAF-inhibitors

**DOI:** 10.1038/s41598-019-40818-w

**Published:** 2019-03-13

**Authors:** Francesca Coperchini, Laura Croce, Marco Denegri, Oriana Awwad, Samuel Tata Ngnitejeu, Marina Muzza, Valentina Capelli, Francesco Latrofa, Luca Persani, Luca Chiovato, Mario Rotondi

**Affiliations:** 1 Unit of Internal Medicine and Endocrinology, ICS Maugeri I.R.C.C.S., Laboratory for Endocrine Disruptors and Chair of Endocrinology University of Pavia, 27100 Pavia, Italy; 2 PHD course in Experimental Medicine, University of Pavia, 27100 Pavia, Italy; 3 Molecular Cardiology, ICS-Maugeri, 27100 Pavia, Italy; 4 Department of Biopharmaceutics and Clinical Pharmacy, The University of Jordan, Amman, 11937 Jordan; 5 Department of General and Minimally Invasive Surgery, ICS Maugeri I.R.C.C.S, Pavia, 27100 Italy; 6 Division of Endocrinology and Metabolism IRCCS Istituto Auxologico Italiano, 20149 Milan, Italy; 7 Department of Clinical and Experimental Medicine, University of Pisa, Pisa, 56124 Italy; 8 Department of Clinical Sciences and Community Health, University of Milan, Milano, 20122 Italy

## Abstract

CXCL8 is a chemokine secreted by normal and thyroid cancer cells with proven tumor-promoting effects. The presence of BRAFV600E mutation is associated with a more aggressive clinical behavior and increased ability to secrete CXCL8 by papillary-thyroid-cancer cells. Aim of this study was to test the effect of the BRAF-inhibitor (PLX4720) on the basal and TNF-α-induced CXCL8 secretions in BRAFV600E mutated (BCPAP, 8305C, 8505C), in RET/PTC rearranged (TPC-1) thyroid-cancer-cell-lines and in normal-human-thyrocytes (NHT). Cells were incubated with increasing concentrations of PLX4720 alone or in combination with TNF-α for 24-hours. CXCL8 concentrations were measured in the cell supernatants. PLX4720 dose-dependently inhibited the basal and the TNF-α-induced CXCL8 secretions in BCPAP (F: 14.3, *p* < 0.0001 for basal and F: 12.29 *p* < 0.0001 for TNF-α), 8305C (F: 407.9 p < 0.0001 for basal and F: 5.76 p < 0.0001 for TNF-α) and 8505C (F:55.24 p < 0.0001 for basal and F: 42.85 p < 0.0001 for TNF-α). No effect was found in TPC-1 (F: 1.8, p = 0.134 for basal; F: 1.6, p = 0.178 for TNF-α). In NHT an inhibitory effect was found only at the highest concentration of PLX4720 (F: 13.13 p < 0.001 for basal and F: 2.5 p < 0.01 for TNF-α). Cell migration assays showed that PLX4720 reduced both basal and CXCL8-induced cell migration in BCPAP, 8305C, 8505C and NHT but not in TPC-1 cells. These results constitutes the first demonstration that PLX4720 is able to inhibit the secretion of CXCL8 in BRAFV600E mutated thyroid cancer cells indicating that, at least some, of the anti-tumor activities of PLX4720 could be exerted through a lowering of CXCL8 in the thyroid-cancer-microenvironment.

## Introduction

BRAF mutation leading to V600E amino acid substitution is identified in the majority of melanomas^1,2^. BRAFV600E mutation is also the most frequent genetic lesion in papillary thyroid cancer (PTC), being present in nearly 40% of cases^3–6^. The presence of BRAFV600E mutation was reported to be associated with a more aggressive clinical behavior of papillary thyroid cancer (PTC)^3,6–9^. The V600E mutation strongly enhances BRAF kinase activity by inserting a negatively charged residue adjacent to the phosphorylation site at T598 and mimicking phosphorylation at Thr598 and Ser601 residues^1,10,11^, with the final result of increasing ERK1/2 phosphorylation^1^. The BRAFV600E oncoprotein confers constitutive activation to the MAPK pathway, which accelerates tumor growth in experimental models of thyroid cancer^12^.

Thyroid cancer microenvironment is composed of a mixture of immune cells and soluble mediators, which are present within and surrounding primary thyroid tumors^13–15^. Among soluble mediators, specific chemokines not only attract different types of immune cell into the tumor site, but also produce pro-tumorigenic actions, including pro-angiogenetic, cyto-proliferative and pro-metastatic effects^13–15^. Thus, the composition of thyroid cancer microenvironment, and in particular the levels of CXCL8 are currently regarded as a key factor for driving tumor progression^16^. In this scenario, no previous study investigated the role of BRAFV600E oncogene in influencing the production of CXCL8 within thyroid cancer microenvironment. Our group recently demonstrated that the BCPAP thyroid cancer cell line, harboring the BRAFV600E mutation, does secrete high amounts of CXCL8 both in basal culture conditions and after incubation with TNFα^17^. CXCL8 has well-known pro-tumorigenic effects in several human cancers including the thyroid ones^16,18^ and its targeting was shown to hamper tumor progression^18,19^. Indeed, *in vitro* and *in vivo* studies demonstrated that CXCL8 plays a crucial role in promoting epithelial-mesenchimal transition (EMT) and migration/metastatization of thyroid cancer cells^20,21^. Supporting these actions, the administration of recombinant CXCL8 in xenografted mice with PTC significantly increased mortality^19,22,23^ while targeting of CXCL8 with an anti-CXCL8 monoclonal antibody significantly prolonged survival^19^. Previous attempts to inhibit CXCL8 secretion in normal and neoplastic thyroid cells were only partially effective owing to the presence of multiple intracellular pathways leading to CXCL8 secretion. Thus, lowering CXCL8 concentrations in thyroid cancer microenvironment requires specific strategies depending on the specific oncogenic background of neoplastic cells^22,24,25^.

Pharmacological compounds with BRAF kinase blocking activity were shown to inhibit the secretion of CXCL8 in melanoma cell lines harboring the BRAFV600E mutation^2^, but their effect in thyroid cancer cells remains to be investigated. Among them, the Plexxikon compound PLX4720 (7-azaindole derivative) is an orally administrable selective inhibitor of BRAFV600E with proven *in vitro* and *in vivo* therapeutic efficacy in melanoma models^26–28^. As for the thyroid, PLX4720 was shown to inhibit the proliferation of BRAFV600E mutated thyroid cancer cell lines *in vitro*^29^ and to reduce tumor growth and distant metastases in a human thyroid cancer model in mice^30^. Whether this BRAF-inhibitor also exerts a CXCL8-lowering effect,in thyroid cancer cells remains to be investigated. Aim of the present study was to test the effect of PLX4720 on the secretion of CXCL8 in normal human thyroid (NHT) cells, in the BCPAP, 8305C and 8505C thyroid cancer cell line harboring the BRAF V600e mutation and in the TPC-1 thyroid cancer cell line bearing the RET/PTC re-arrangement. The potential effect of PLX4720 on the basal and CXCL8-induced thyroid cell migration was also investigated.

## Results

### Effects of treatment with PLX4720 in terms of inhibition of CXCL8 secretion in thyroid cancer cell lines and NHT

**Figure 1:**
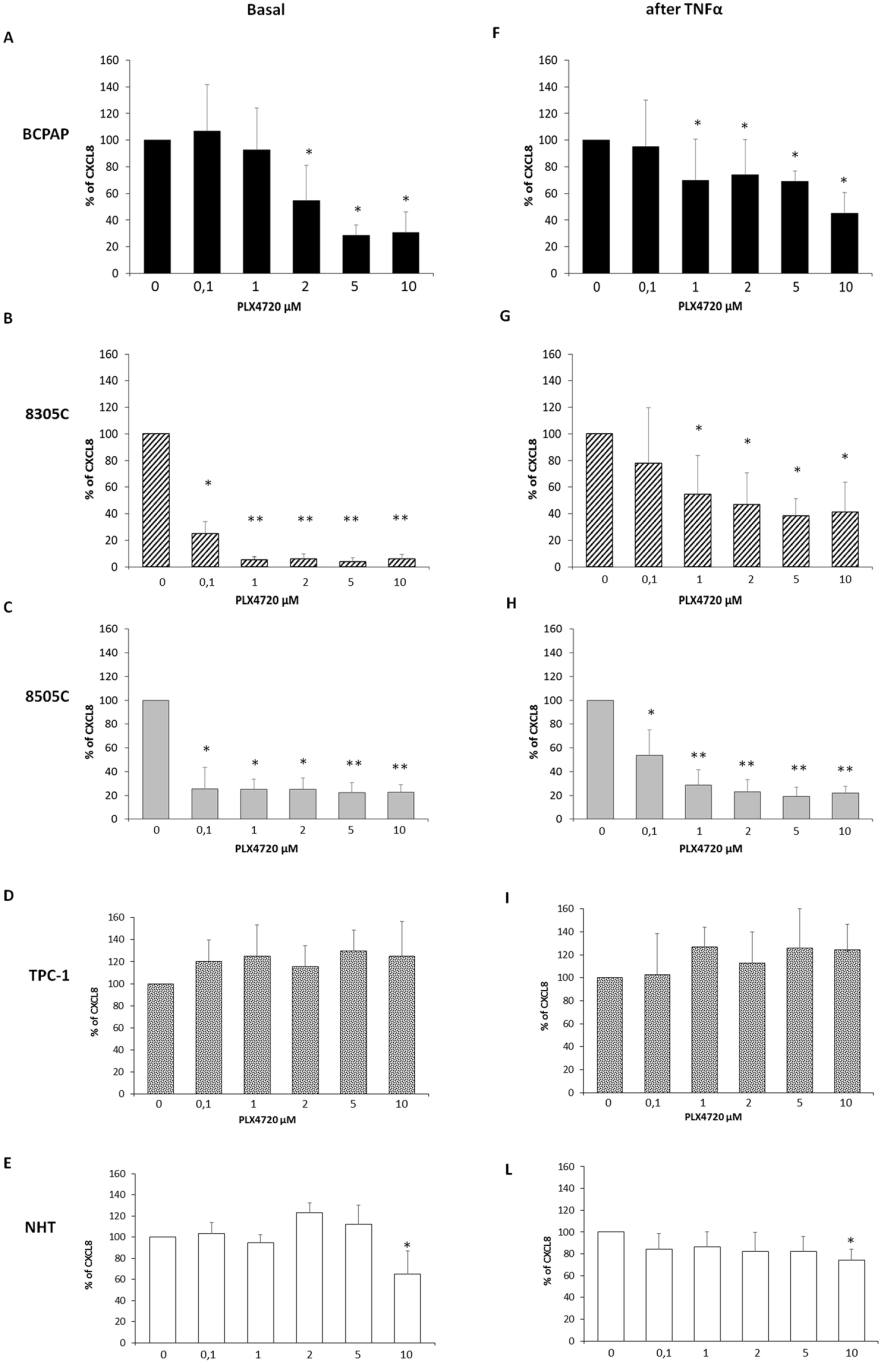
Panel A PLX4720 inhibit the basal CXCL8 secretion in BCPAP (ANOVA F: 14.3; p < 0.0001), the inhibitory effect was significant starting by 2 µM concentration (Post Hoc analysis by Bonferroni *p < 0.001 vs. basal). Panel B PLX4720 inhibited the basal CXCL8 secretion in 8305C (ANOVA F: 407.9; p < 0.0001) (ANOVA F: 407.9; p < 0.0001), the inhibitory effect was significant starting from 0.1 µM (Post Hoc analysis by Bonferroni *p < 0.001 vs. basal, **p < 0.001 vs. 0,1 µM). Panel C PLX4720 inhibited the basal CXCL8 secretion in 8505C (ANOVA F: 55.24; p < 0.0001), the inhibitory effect started from 0.1 µM (Post Hoc analysis by Bonferroni *p < 0.001 vs. basal, p < 0.001 vs. 0.1 µM). Panel D Basal secretion of CXCL8 was not inhibited by PLX4720 in TPC-1 cell lines at any concentrations (ANOVA F: 1.8, p = 1.34). Panel E Basal secretion of CXCL8 was inhibited by PLX4720 in NHT (ANOVA F: 13.13; *p* < 0.001) being significant only at the higher concentration of 10 µM (*Post Hoc* analysis by Bonferroni *p < 0.01 *vs*. basal). Panel F PLX4720 inhibit the TNF-𝛼-stimulated CXCL8 secretion in BCPAP (ANOVA F: 12.29 *p* < 0.0001), the inhibitory effect was significant starting by 1 10 µM (3.13; t an*Post Hoc* analysis by Bonferroni *p < 0.001 vs. TNF-𝛼). Panel G PLX4720 inhibit the TNF-*α*-stimulated CXCL8 secretion in 8305C (ANOVA F: 5.76 p < 0.0001), the inhibitory effect started from 1 rom *Post Hoc* analysis by Bonferroni *p < 0.001 vs. TNF-𝛼). Panel H PLX4720 inhibit the TNF-𝛼-stimulated CXCL8 secretion in 8505C (ANOVA F: 42.85 p < 0.0001), the inhibitory effect started from 0.1 rom *Post Hoc* analysis by Bonferroni *p < 0.001 vs. TNF-𝛼, **p < 0.001 vs. 0.1 ni *p < Panel I TNF-𝛼-stimulated CXCL8 secretion was not inhibited by PLX4720 in TPC-1 cell lines at any concentrations (ANOVA F: 1.6, p = 1.78). Panel E TNF-𝛼-stimulated CXCL8 secretion was inhibited by PLX4720 in NHT (ANOVA F: 2.5; *p* < 0.01) being significant only at the higher concentration of10 µM (*Post Hoc* analysis by Bonferroni *p < 0.001 *vs*. TNF-α alone).

CXCL8 concentrations were assayed in the supernatants of, BCPAP, 8305C and 8505C in basal and TNF-𝛼-stimulated condition. As previously reported, TNF-𝛼 elicited a significant increase in the concentrations of CXCL8 in the supernatants of BCPAP^17,31^ but also in 8305C and 8505C cells (data not shown). The treatment with PLX4720 significantly and in a dose-dependent manner, inhibited the secretion of CXCL8 in: BCPAP (ANOVA F: 14.3; p < 0.0001) (Fig. 1A), in 8305C (ANOVA F: 407.9; p < 0.0001) (Fig. 1B) and in 8505C (ANOVA F: 55.24; p < 0.0001) (Fig. 1C). *Post Hoc* analysis performed by Bonferroni demonstrated some differences in the strength of inhibition induced by PLX4720 in different cell types. Indeed, significant inhibition of the basal secretion of CXCL8 started from a 2 µM concentration of PLX4720 in BCPAP (p < 0.001 vs. basal) (Fig. 1A) and from a 0.1 µM concentration of PLX4720 in 8305C (Fig. 1B) and 8505C (Fig. 1C); (p < 0.001 vs. basal for both 8305C and 8505C).

PLX4720 also produced a significant and dose-dependent inhibition of the TNFα-induced CXCL8 secretion in BCPAP cells (ANOVA F: 12.29 *p* < 0.0001) (Fig. 1F), in 8305C cells (ANOVA F: 5.76 p < 0.0001) (Fig. 1G) and in 8505C cells (ANOVA F: 42.85 p < 0.0001) (Fig. 1H). Also in this case, some differences among cell types were observed. Indeed, *Post Hoc* analysis by Bonferroni showed that the CXCL8 inhibiting effect of PLX4720 was significant starting from 1 µM in BCPAP (Fig. 1F) and in 8305C (Fig. 1G) (p < 0.001 vs. TNF-𝛼 alone for both cells**)** while in 8505C, a significant inhibition of the TNF-𝛼-stimulated CXCL8 secretion started from a 0.1 µM concentration of PLX4720 (p < 0.001 vs. TNF-𝛼 alone) (Fig. 1H).

At difference with the above findings, PLX4720 did not produce any effect in terms of CXCL8 inhibition on TPC-1 cell lines, both in basal (ANOVA F: 1.8; p = 0.134) and in TNF-𝛼-stimulated conditions (ANOVA F: 1.6; p = 0.178) (Fig. 1D–I). However, treatment with PLX4720 inhibited both the basal (ANOVA F: 13.13; *p* < 0.001) and the TNF-𝛼-stimulated CXCL8 secretion (ANOVA F: 2.5; *p* < 0.01) in NHT (Fig. 1E–L). *Post Hoc* analysis by Bonferroni evidenced a significant inhibition of the basal and the TNF-𝛼-stimulated CXCL8 secretion only at the highest (10 µM) PLX4720 concentration (p < 0.01 *vs*. basal; p < 0.001 *vs*. TNF-α alone) (Fig. 1E–L).

Overall, the inhibition of CXCL8 secretion was paralleled by the inhibition of ERK phosphorylation, as assessed by Western blot, in all cell types with the exception of TPC-1 cells in which no CXCL8 inhibiting effect and no reduction of ERK phosphorylation was found (Supplemental Fig. S1).

### Time course of PLX4720 inhibition of CXCL8 secretion

**Figure 2:**
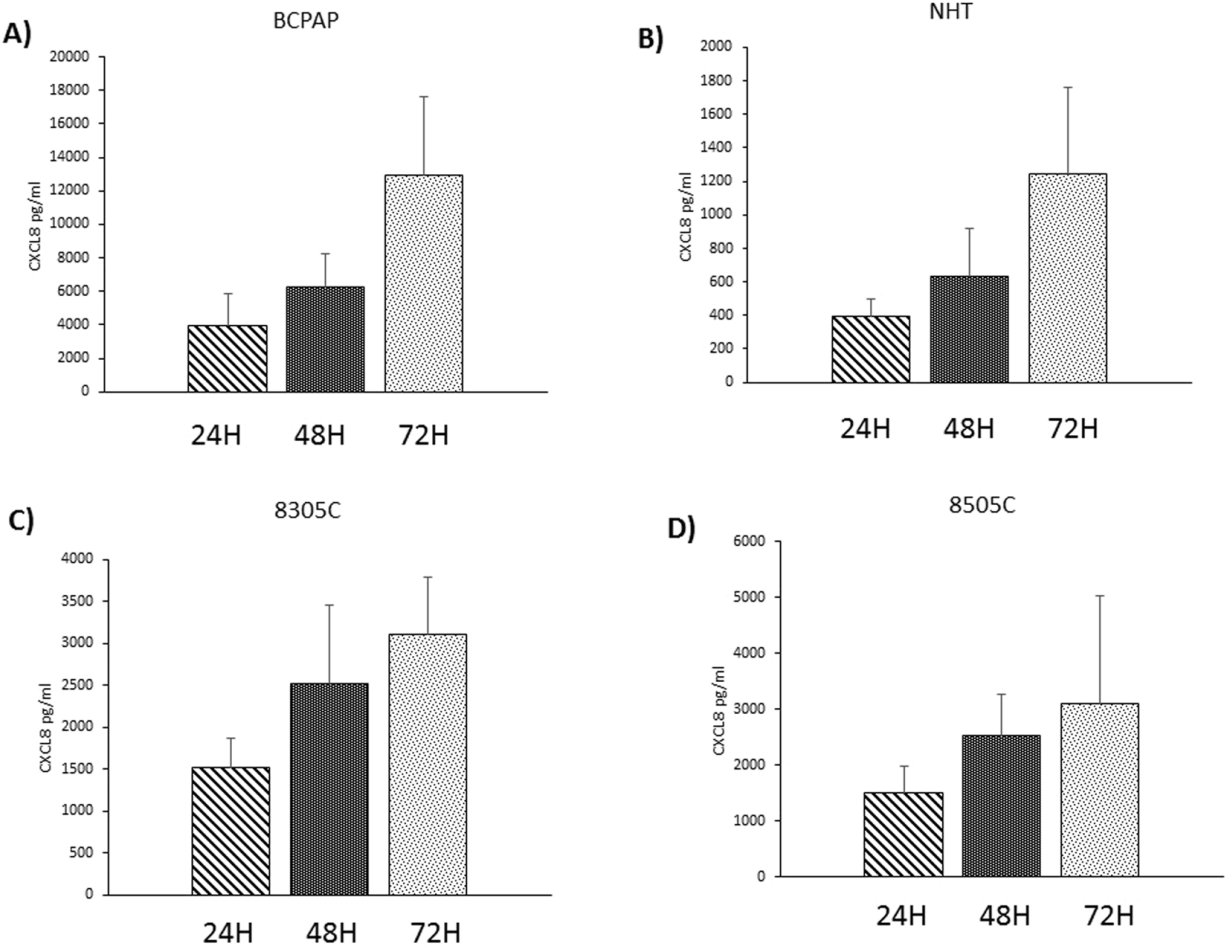
CXCL8 concentrations progressively increased throughout the 72-h time course in untreated thyroid cells. Panel A BCPAP cells (ANOVA: F = 13.39; p < 0.0001). Panel B NHT cells (ANOVA: F = 2,7; p < 0.002). Panel C 8305C cells (ANOVA F = 6.76; p < 0.009). Panel D 8505C cells (ANOVA: F = 11.09; p < 0.001).

**Figure 3:**
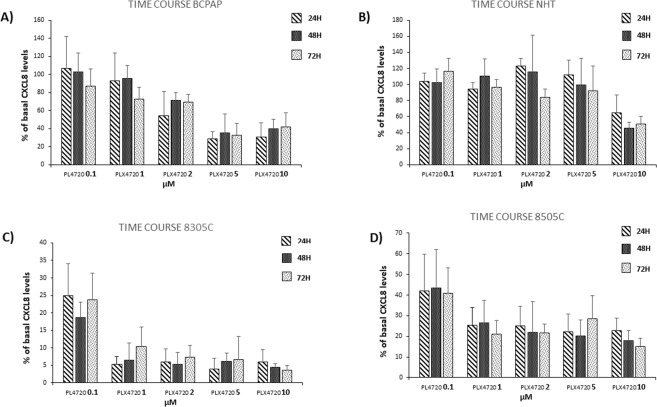
Time course of PLX4720 inhibitory effect on CXCL8 secretion. No significant change in the inhibitory power of PLX4720 at a given concentration was found throughout the 72-h time course. Panel A BCPAP (ANOVAs: PLX4720 0.1 µM F: 0.6; NS; 1 µM F: 1.7; NS; 2 µM F: 1.7; p = 0.2; 5 µM F: 2.7; p = 0.7; 10 µM F: 1.0, NS). Panel B NHT (ANOVAs: PLX4720 0.1 µM F:1.7, NS; 1 µM F:2.8; NS; 2 µM F: 3; NS; 5 µM F: 1; NS; 10 µM F: 2.3, NS). Panel C 8305C (ANOVAs: PLX4720 0.1 µM F:1.6, NS; 1 µM F: 2.6; NS; 2 µM F: 0.5; NS; 5 µM F: 0.4; NS; 10 µM F: 1.3, NS). Panel D 8505C (ANOVAs: PLX4720 0.1 µM F:0.02, NS; 1 µM F: 0.2; NS; 2 µM F: 0.1; NS; 5 µM F: 1.2; NS; 10 µM F: 2.9, NS).

To assess whether the CXCL8-inhibiting effect of PLX4720 could vary over time, a time course of CXCL8 secretion following treatment with PLX4720 was performed. For this set of experiments those cells in which an inhibitory effect on CXCL8 secretion was exerted by PLX4720 (NHT, BCPAP, 8505C and 8305C) were treated with PLX4720 (0, 0.1, 1, 2, 5, 10 µM) for 24, 48 and 72 hours. A first finding was that, throughout the time course the basal levels of CXCL8 progressively increased in all cell types (although at a different magnitude) (ANOVAs: F = 13.39; p < 0.0001 for BCPAP; F = 6.76; p < 0.009 for 8305C; F = 11.09; p < 0.001 for 8505C; F = 2,7; p < 0.002 for NHT) **(**Fig. 2A–D). The percentages of inhibition of the CXCL8 secretion were compared for each concentration of PLX4720 at 24-h, 48-h and 72-h in each cell type. Separated ANOVAs for each cell type were performed to assess possible differences in percentages of inhibition of CXCL8 secretion by PLX4720 at each concentration throughout the time course. As shown in Fig. 3(A–D), no significant change in the inhibitory power of a given concentration of PLX4720 as assessed at 24 h, 48 h and 72 h could be found in any cell type. These findings would suggest that the CXCL8-inhibitory effect of PLX4720 is maintained (at a similar strength of inhibition) up to 72 hours.

### Migration Assays

To assess whether the PLX4720-mediated inhibition of CXCL8 secretion would produce any biological consequence, cell migration assays were performed.

**Figure 4:**
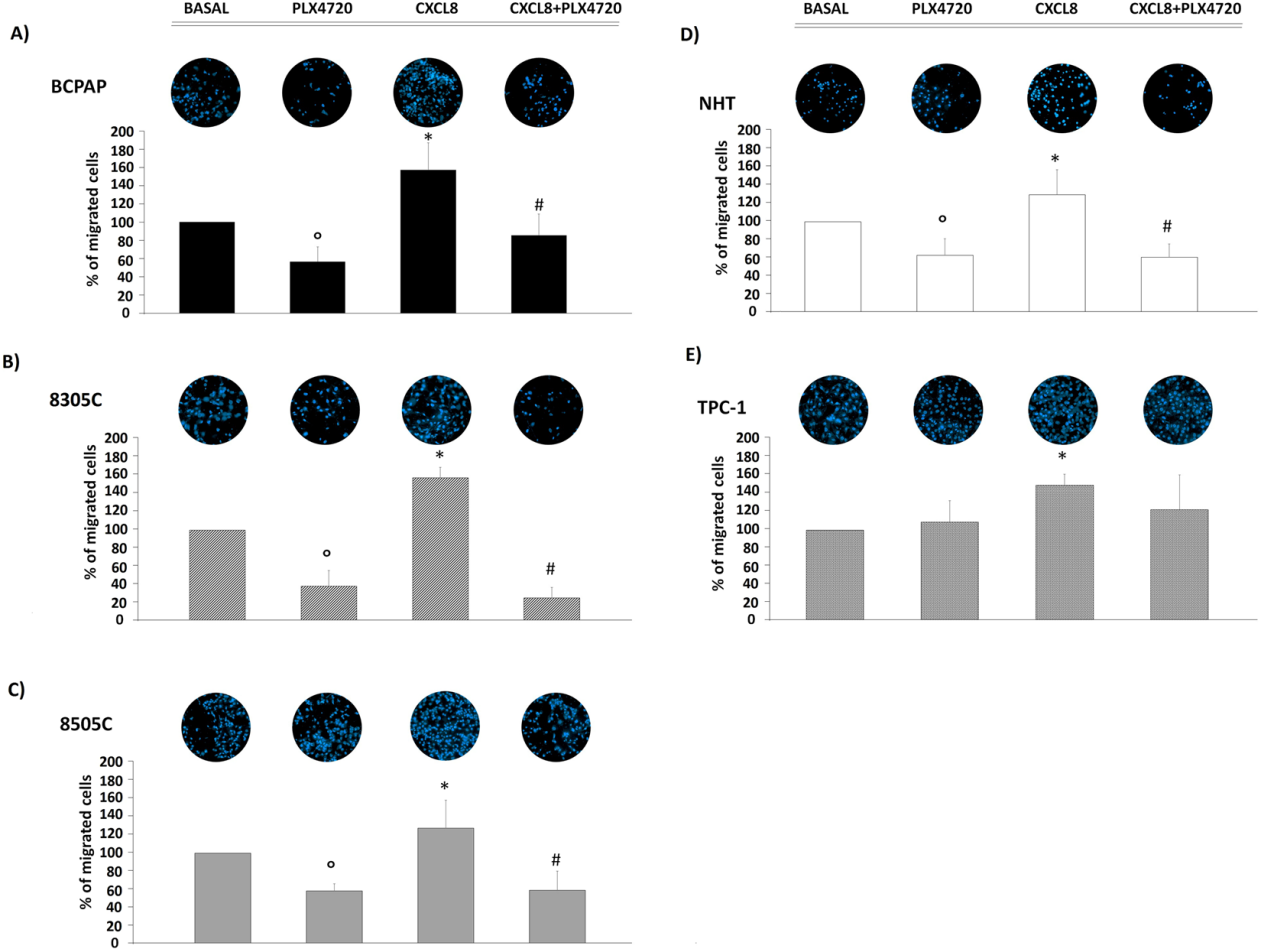
Effect of PLX4720 on the basal and rh-CXCL8-induced migration in BCPAP, 8305C, 8505C, NHT and TPC-1 thyroid cancer cells. Representative images and the respective histograms after 16 hours of migration within the trans-well migration chamber system in BCPAP, 8305C, 8505C, NHT and TPC-1 (Panels A–E respectively). Panel (A) in BCPAP the treatment with PLX4720 10 µM reduced the basal migration (ANOVA F = 27.9, p < 0.0001; *Post Hoc* °p < 0.005 vs. basal). The incubation of BCPAP with rh-CXCL8 produced a significant increase of BCPAP migration (*Post Hoc* *p < 0.0001 vs. basal). The co-incubation with rh-CXCL8 and PLX4720 10 µM significantly inhibited the BCPAP migration induced by rh-CXCL8 (*Post Hoc* ^#^p < 0.001 vs. CXCL8). Panel (B) in 8305C, the treatment with PLX4720 10 µM reduced the basal migration of 8305C (ANOVA F = 161.7, p < 0.0001; *Post Hoc* °p < 0.005 vs. basal). The incubation of 8305C with rh-CXCL8 produced a significant increase of 8305C migration (*Post Hoc* *p < 0.0001 vs. basal). The co-incubation with rh-CXCL8 and PLX4720 10 µM significantly inhibited the 8305C migration induced by rh-CXCL8 (*Post Hoc* ^#^p < 0.0001 vs. CXCL8). Panel (C) in 8505C the treatment with PLX4720 10 µM reduced the basal migration of 8505C (ANOVA F = 21.6, p < 0.0001; *Post Hoc* °p < 0.005 vs. basal). The incubation of 8505C with rh-CXCL8 produced a significant increase of 8505C migration (*Post Hoc* *p < 0.0001 vs. basal). The co-incubation with rh-CXCL8 and PLX4720 10 µM significantly inhibited the 8505C migration induced by rh-CXCL8 (*Post Hoc* ^#^p < 0.0001 vs. CXCL8). Panel (D) in NHT, the treatment with PLX4720 10 µM reduced the basal migration of NHT (ANOVA F = 25.3, p < 0.0001; *Post Hoc* °p < 0.005 vs. basal). The incubation of NHT with rh-CXCL8 produced a significant increase of NHT migration (*Post Hoc* *p < 0.05 vs. basal). The co-incubation with rh-CXCL8 and PLX4720 10 µM significantly inhibited the NHT migration induced by rh-CXCL8 (*Post Hoc* ^#^p < 0.0001 vs. CXCL8). Panel (E) in TPC-1 the treatment with PLX4720 10 µM did not reduce the basal migration of TPC-1 (ANOVA F = 4.4, p < 0.01), *Post Hoc* NS vs. basal). The incubation of TPC-1 with rh-CXCL8 produced a significant increase of TPC-1 migration (*Post Hoc* *p < 0.05 vs. basal). The co-incubation with rh-CXCL8 and PLX4720 10 µM did not reduce the TPC-1 migration induced by rh-CXCL8 (*Post Hoc* # NS vs. CXCL8). Bar graphs show the corresponding analysis of migrated cells on the lower side of the transwell filter. Basal migration was conventionally estimated as 100%.

The transwell migration assay showed that rh-CXCL8 induced migration in all cell types. In details, the following results were found: 150 ± 20% migrated cells in BCPAP (ANOVA F = 27.9 p < 0.0001; *Post Hoc* CXCL8 vs. basal p < 0.0001), 150 ± 0.1% migrated cells in 8305C (ANOVA F = 161.7 p < 0.0001; *Post Hoc* CXCL8 vs. basal p < 0.0001), 120 ± 30% migrated cells in 8505C (ANOVA F = 21.6 p < 0.0001; *Post Hoc* CXCL8 vs. basal p < 0.05), 130 ± 20% migrated cells in NHT, (ANOVA F = 25.3 p < 0.0001; *Post Hoc* CXCL8 vs. basal p < 0.05), 140 ± 10% migrated cells in TPC-1, (ANOVA F = 4.4 p < 0.01; *Post Hoc* CXCL8 vs. basal p < 0.05) Fig. 4(A–E). The incubation with PLX4720 inhibited basal cell migration of BCPAP (mean: 56 ± 10% migrated cells; *Post Hoc* PLX4720 *vs* basal p < 0.005), 8305C (mean: 57 ± 8% migrated cells; *Post Hoc* PLX4720 *vs* basal p < 0.0001), 8505C (mean: 37 ± 17% migrated cells; *Post Hoc* PLX4720 *vs* basal p < 0.005) and NHT (mean: 62 ± 18% migrated cells; *Post Hoc* PLX4720 *vs* basal p < 0.005) Fig. 4(A–D). On the other hand, PLX4720 did not inhibit the basal migration of TPC-1 (mean: 108 ± 24% migration; *Post Hoc* PLX4720 *vs* basal NS) Fig. 4E. Finally, co-treatment with PLX4720 and rh-CXCL8 had an inhibitory effect on the CXCL8-induced cell migration of BCPAP (mean: 85 ± 20% migrated cells; *Post Hoc* CXCL8+ PLX4720 *vs* CXCL8 p < 0.001), 8305C (mean: 24 ± 10% migration; *Post Hoc* CXCL8+ PLX4720 *vs* CXCL8 p < 0.0001) 8505C (mean: 58 ± 20% migration; *Post Hoc* CXCL8+ PLX4720 *vs* CXCL8 p < 0.0001) and NHT (mean: 60 ± 10% migration; *Post Hoc* CXCL8+ PLX4720 *vs* CXCL8 p < 0.0001) but not in TPC-1 cells (mean: 120 ± 30% migration; *Post Hoc* CXCL8+ PLX4720 *vs* CXCL8 NS). Figure 4(A–E).

As a further confirmation of these findings, wound healing assay was performed (Supplemental Fig. S2).

## Discussion

The results of the present study show that the selective BRAF-inhibitor, PLX4720, inhibits the basal and the TNFα-stimulated secretion of CXCL8 in BRAFV600E mutated thyroid cancer cell lines (BCPAP, 8305C and 8505C). The inhibitory effect of PLX4720 was observed also in primary cultures of NHT, even if only at the maximal concentration. On the other hand, PLX4720 did not produce any significant effect in terms of CXCL8 inhibition in TPC-1 cells harboring RET-PTC rearrangement.

It should be highlighted that, the strength of the inhibitory effect of PLX4720 (both in terms of minimal concentration able to elicit a significant inhibitory effect and in terms of percentages of inhibition) was different in relation to the specific cell type. Indeed, the CXCL8-inhibiting effect of PLX4720 was stronger in 8305C and 8505C tumor cell lines as compared to BCPAP which would fit with the notion that not all tumors may show the same effects from a single targeted agent^32^. The finding that the inhibitory effect of PLX4720 on CXCL8 secretion was not limited to BRAFV600E mutated cells, but also occurred in NHT could be regarded as unexpected and deserves to be discussed.

The fact that the concentrations of CXCL8 in the supernatants of NHT cells treated with PLX4720 progressively increased up to 72 hours of culture and that the percentage of inhibition versus untreated NHT cells remained constant would not support the hypothesis of a cytotoxic effect exerted by PLX4720. The issue of a potential cytotoxic effect of PLX4720 was addressed by previous studies on both normal and thyroid cancer cells demonstrating lack of any cytotoxic effect^29,33^. In particular, Nucera *et al*. demonstrated that PLX4720 treatment (1 µM or 10 µM which corresponds to the concentrations used in the present study) did not lead to apoptosis as assessed by flow cytometric analysis^33^. Taken together these data indicate that the inhibition of CXCL8 in NHT cells is not due to a cytotoxic effect of PLX4720. A more likely explanation stems from the study by Tsai *et al*., who demonstrated that PLX4720 does inhibits wildtype BRAF kinase activity in several tumor cell lines, but only at higher concentrations than those effective on cells harboring a mutated BRAFV600E kinase^27^. These data provide an explanation for the high concentration of PLX4720 required to produce a significant inhibition of the CXCL8 in NHT cells. The inhibitory effect of PLX4720 on the secretion of CXCL8 by NHT cells should not be underestimated because these cells are by far the most abundant ones in a neoplastic thyroid, being their contribution to the global secreted amounts of CXCL8 in the tumor microenvironment not negligible^25^.

A further aspect deserving to be commented is that the inhibitory power of PLX4720 (expressed as % of inhibition) for a given cell type and a given concentration of PLX4720 was maintained at a similar degree over a 72-h time course indicating lack of any rebound effect of the CXCL8 inhibiting effect. The existence of a rebound effect was previously demonstrated by Montero-Conde C *et al*., showing that the effects of BRAF inhibition in BRAF mutant thyroid cancer cells, were hampered (after 72 hours) by the release of negative feedback mechanisms^34^.

PLX4720 was previously demonstrated to exert several anticancer effects in both xenografts models of melanoma and thyroid cancer. In animal models of melanoma, treatment with PLX4720 leads to a reduction of tumor growth and induces a higher expression of apoptotic genes and cell cycle arrests in neoplastic cells^26,27^.

As far as thyroid tumors are concerned, treatment with PLX4720 lead to a reduction of proliferation, migration and invasion of thyroid cancer cells^29,33^.

In this regards, the results of the present study suggest that at least some of the anti-cancer effect of PLX4720 might be exerted also through a reduction of CXCL8 within the tumor microenvironment. Indeed, even if CXCL8 inhibition might not be the only mechanism, the results of migration assays experiments clearly indicated that PLX4720 was able to reduce the migration of thyroid cells. In particular, this effect was evident only in those cells in which the treatment by PLX4720 also produced an inhibition of the CXCL8 secretion (BCPAP, 8305C, 8505C, NHT) but not in those cells in which PLX4720 did not inhibit CXCL8 secretion (TPC-1). These results highlight that the ability of PLX4720 in inhibiting CXCL8 secretion would in turn, result in potentially relevant biological consequences.

The results of the present study constitute the first demonstration of a CXCL8-inhibiting effect exerted by PLX4720 in thyroid cancer cells and should be regarded as potentially relevant. Indeed, CXCL8 is a chemokine with extensively described pro-tumorigenic effects which include influencing of tumor cell growth, angiogenesis, invasiveness and EMT^16,20,35,36^. Furthermore targeting/lowering of CXCL8 levels is known to produce beneficial effects in thyroid cancer. Interestingly, a previous *in vivo* study showed that patients with melanoma harboring the BRAFV600E mutation experience a significant decrease in the circulating levels of CXCL8 while treated with BRAF-inhibitors^2^.

In this view, the present study was specifically aimed at evaluating whether PLX4720 (already proven to have several anti cancer effects) was also able to inhibit CXCL8 secretion which could be regarded “*per se*” as beneficial against cancer. In conclusion, the here reported results, would suggest a further anti-cancer effect of PLX4720 which is manifested by its ability to reduce CXCL8 secretion. Although *in vivo* studies will be required to assess the therapeutic benefits deriving from the inhibition of CXCL8 secretion in thyroid tumor microenvironment, this could represent an alternative therapeutic strategy, at least for those minority of patients who are refractory to conventional therapies.

## Materials and Methods

### Primary cultures of NHT

Surgical specimens of normal human thyroid were obtained from the contralateral disease-free lobe of patients who underwent thyroidectomy for a solitary non-functioning nodule (n = 3). The study was approved by the Institutional Board of ICS-Maugeri. Before surgery, written informed consent to the study was obtained from all patients. All the experiments were performed in accordance with the relevant guidelines and regulations. Surgical specimens were minced and then incubated with collagenase type II (Sigma, Saint Louis, MO, USA) 5 mg/ml, in 5 ml of Coon’s F12 medium, for 4 h at 37 °C as previously described^37^. Then, 10 ml of Coon’s F12 medium were added, following which, cells were filtered, spun at 1000 × g for 10 min, washed with Coon’s F12 medium, spun again, and finally re-suspended in complete medium containing 5% newborn calf serum and a mixture of six hormones including insulin (5 μg/ml), hydrocortisone (50 μg/ml), transferrin (5 μg/ml), somatostatin (10 ng/ml), gly-his-lysine (10 ng/ml) and bovine TSH (1 mU/ml).

### Thyroid tumor cell lines BCPAP, TPC-1, 8305C and 8505C

Human thyroid cancer cell lines, BCPAP harboring the BRAF V600E mutation and TPC-1 bearing the RET/PTC rearrangement, were a gift of Prof. M. Santoro (Medical School, University “Federico II” of Naples, Naples, Italy). These cell lines had been previously tested and authenticated by DNA analysis. Cancer cells were propagated in Dulbecco’s Modified Eagle Medium (DMEM) (Sigma, Saint Louis, MO, USA) supplemented with 10% fetal bovine serum (Sigma, Saint Louis, MO, USA), 2 mM L-glutamine and 100 U/ml penicillin/streptomycin (Sigma, Saint Louis, MO, USA) as previously described^37^. Human thyroid cancer cell lines, 8305C and 8505C harboring the BRAF V600E mutation were previously tested and authenticated by DNA analysis. 8305C cells were propagated in Mininum Essential Medium (MEM) (Sigma, Saint Louis, MO, USA) supplemented with 10% fetal bovine serum (Sigma, Saint Louis, MO, USA), 2 mM L-glutamine and 100 U/ml penicillin/streptomycin (Sigma, Saint Louis, MO, USA). 8505C cells were propagated in RPMI medium (Sigma, Saint Louis, MO, USA) supplemented with 10% fetal bovine serum (Sigma, Saint Louis, MO, USA), 2 mM L-glutamine and 100 U/ml penicillin/streptomycin (Sigma, Saint Louis, MO, USA). Cells were incubated with the chosen stimuli in serum-free medium.

### Basal and TNF-α-induced CXCL8 secretion in BCPAP, 8305C, 8505C, TPC-1 thyroid cancer cell lines and in primary cultures of NHT in the presence or absence of increasing concentrations of PLX4720

For the CXCL8 secretion assays, 3000 cells were seeded into 96-well plates in complete medium. After adherence to the plastic surface, BCPAP, TPC-1, 8305C, 8505C and NHT cells were incubated for 24 h in serum-free medium (basal condition) with increasing concentrations of PLX4720 (0, 0.1, 1, 2, 5, 10 µM) (Sigma Aldrich). Incubation with PLX4720 was done in the absence (basal secretion) and in the presence (stimulated condition) of TNF-α 10 ng/ml. All experiments were performed in triplicates.

### Time course of PLX4720 inhibition of CXCL8 secretion in BCPAP, 8305C, 8505C, TPC-1 thyroid cancer cell lines and in primary cultures of NHT

For the CXCL8 secretion assays, 3000 cells were seeded into 96-well plates in complete medium. After adherence to the plastic surface, BCPAP, 8305C, 8505C and NHT cells were incubated for 24, 48 and 72 hours with or without PLX4720 0, 0.1, 1, 2, 5, 10 µM. All experiments were performed in triplicates.

### ELISA for CXCL8

CXCL8 was measured in cell supernatants of BCPAP, TPC-1, 8305C, 8505C and NHT cells using commercially available kits (R&D Systems, Minneapolis, MN). The mean minimum detectable concentration of CXCL8 was 3.5 pg/ml. The intra- and inter-assay coefficients of variation were 3.4% and 6.8%, respectively. Samples were assayed in duplicates.

### Western blot for pERK

Cells were seeded in 6 well plates at a concentration of 500000 cells/well and treated for 24 hours with increasing concentrations of PLX4720. Subsequently cells were washed three times with PBS and incubated 10 minutes in buffer RIPA supplemented with protease inhibitors on ice. Cells were then scraped and centrifuged. The amounts of proteins in the supernatants were quantified using the BCA kit (Thermo Scientific). Western blots were conducted by standard procedure on 15 μg protein. 1:1000 anti-phospho ERK1/2 (αP-ERK1/2) or anti-ERK1/2 antibodies (αERK1/2; Cell Signaling Technology, Inc., Danvers, MA, USA) were used. Bands were quantified by densitometry with the Quantity One program version 4.6.1. (BioRad). (Supplemental materials).

### Migration

The cell migration assay was performed with the trans-well migration chamber system (Merck Millipore, Milan, Italy), as previously described^37^. Briefly, NHT, TPC-1, BCPAP, 8305C and 8505C were cultured for 24 hours with fresh medium alone or supplemented with 10 µM of PLX4720, 100 ng/ml of CXCL8 (R&D Systems, Minneapolis) or their combination. After treatment, 20 × 10^3^ cells/well were seeded in the upper chambers of the 96-well plate with polycarbonate inserts having 0.3Cm^2^/well membrane area and 8 μm pore size. In each condition the lower chambers were filled with 150 μl of the corresponding medium. Cells were left to migrate for 16 hours at 37 °C and 5% CO_2_. At the end of the incubation, samples were analyzed as previously described^38^. Briefly, cell inserts were washed three times with PBS and migrated cells on the underside of the membrane were fixed with 4% paraformaldehyde for 20 minutes. Cell nuclei were then stained with Hoechst 33342 (1:1000) (Life Technologies, Monza, Italy). Finally, the membranes were cut out with a scalpel, and mounted onto glass slides with DACO reagent (Life Technologies, Monza, Italy). Three replicates were evaluated for each condition. Images were acquired using an Olympus BX51 microscope (Olympus, Deutschland GmbH, Hamburg, Germany). The number of migrated cells was counted analyzing 12 random fields of the membranes per condition. Data are expressed as % of mean numbers of migrated cells ± standard deviation (SD).

### Wound-healing assay

For wound-healing assay cells were seeded in a 24-well plate. When cells reached nearly the 90% of cell confluence, a scratch wound was created in the monolayer using a sterile 200 µl pipette tip. Cells were then treated with fresh medium alone, or supplemented with 10 µM of PLX4720. Phase contrast images were captured between 0 and 24 hours using an Olympus IX53 microscope (Olympus, Deutschland GmbH, Hamburg, Germany). Data are expressed as the percentages of the remaining gap area after 24 hours relative to the initial gap area (0 hours). The area was measured using the LCmicro software (Olympus Soft Imaging Solutions GmbH) (Supplemental materials).

### Statistical analysis

Statistical analysis was performed using the SPSS software (SPSS, Inc., Evanston, IL). Mean group values were compared by using one-way ANOVA for normally distributed variables. *Post Hoc* analysis was performed according to the Bonferroni’s correction for multiple comparisons. Values are reported as mean ± SD unless otherwise noted. A *p* value < 0.05 was considered statistically significant.

## Supplementary information

Supporting Information For: The BRAF-inhibitor PLX4720 inhibits CXCL8 secretion in BRAFV600E mutated and normal thyroid cells: a further anti-cancer effect of BRAF-inhibitors

## Data Availability

There are no restrictions on the availability of materials or information.
