# Supplementary material for: The BRAF-inhibitor PLX4720 inhibits CXCL8 secretion in BRAFV600E mutated and normal thyroid cells: a further anti-cancer effect of BRAF-inhibitors
Source: Sci Rep. 2019 Mar 13;9:4390. doi: 10.1038/s41598-019-40818-w (PMC6416278; doi:10.1038/s41598-019-40818-w)
Supplement: Supplementary file 1 — Supporting Information For: The BRAF-inhibitor PLX4720 inhibits CXCL8 secretion in BRAFV600E mutated and normal thyroid cells: a further anti-cancer effect of BRAF-inhibitors [file 41598_2019_40818_MOESM1_ESM.docx]

***Supporting Information For:***

**The BRAF-inhibitor PLX4720 inhibits CXCL8 secretion in BRAFV600E mutated and normal thyroid cells: a further anti-cancer effect of BRAF-inhibitors**

**Francesca Coperchini**^1^**, Laura Croce**^1,2^**, Marco Denegri^3^, Oriana Awwad^4^, Samuel Tata Ngnitejeu^5^, Marina Muzza^6^, Valentina Capelli**^1^**, Francesco Latrofa^7^, Luca Persani^8^, Luca Chiovato**^1^***, Mario Rotondi**^1^

^1^ Unit of Internal Medicine and Endocrinology, ICS Maugeri I.R.C.C.S., Laboratory for Endocrine Disruptors and Chair of Endocrinology University of Pavia, 27100, Italy;

^2^ PHD course in Experimental Medicine, University of Pavia 27100, Italy;

^3^Molecular Cardiology, ICS-Maugeri, 27100, Pavia, Italy

^4^Department of Biopharmaceutics and Clinical Pharmacy, The University of Jordan, Amman 11937, Jordan;

^5^Department of General and Minimally Invasive Surgery, ICS Maugeri I.R.C.C.S., Pavia, 27100, Italy

**^6^**Division of Endocrinology and Metabolism IRCCS Istituto Auxologico Italiano, 20149 Milan, Italy

^7^Department of Clinical and Experimental Medicine, University Of Pisa, Pisa, 56124, Italy.

**^8^** Department of Clinical Sciences and Community Health, University of Milan, Milano, 20122 Italy; Division of Endocrine and Metabolic Diseases, IRCCS Istituto Auxologico Italiano, Milano, 20149, Italy

***** [**luca.chiovato@icsmaugeri.it**](mailto:luca.chiovato@icsmaugeri.it)

**
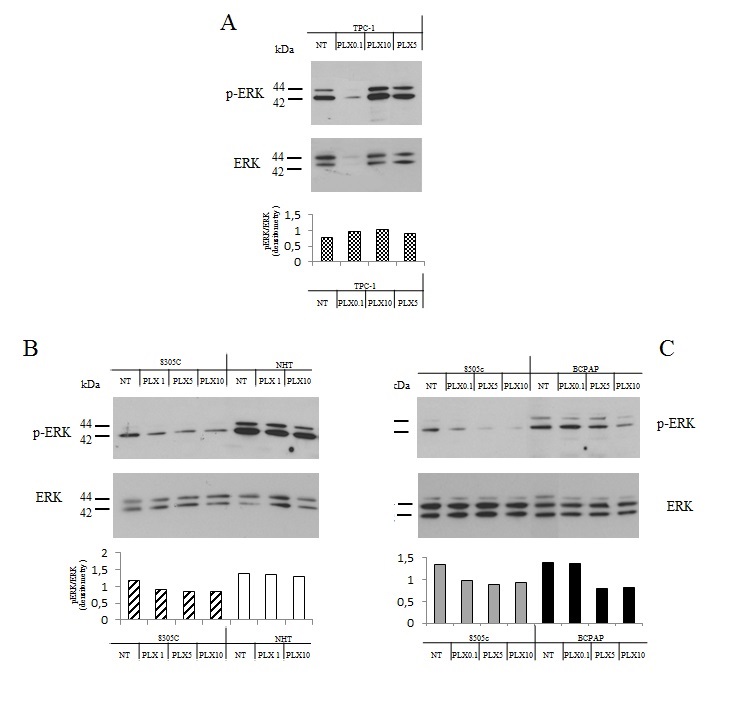
**

**Figure S1:** **ERK phosphorylation**. Total lysates from all cells tested after treatment with different PLX4720 concentrations for 24 hours were probed with anti-phospho ERK (aP-ERK) or anti-ERK antiserum (aERK). The ERK1 (44 kDa) and ERK2 (42 kDa) isoforms are indicated by lines.

No reduction was found in TPC-1 (**panel A**). In 8305C, 8505C the reduction of ERK-phosforilation was found at all chosen concentration of PLX4720 (**panel B and C**). In NHT a lower inhibition seems to occur only at 10 µM (**panel B**). In BCPAP a reduction of ERK phosphorylation was found at 5 and 10µM, but not at 0,1 µM of PLX4720 (**panel C**).


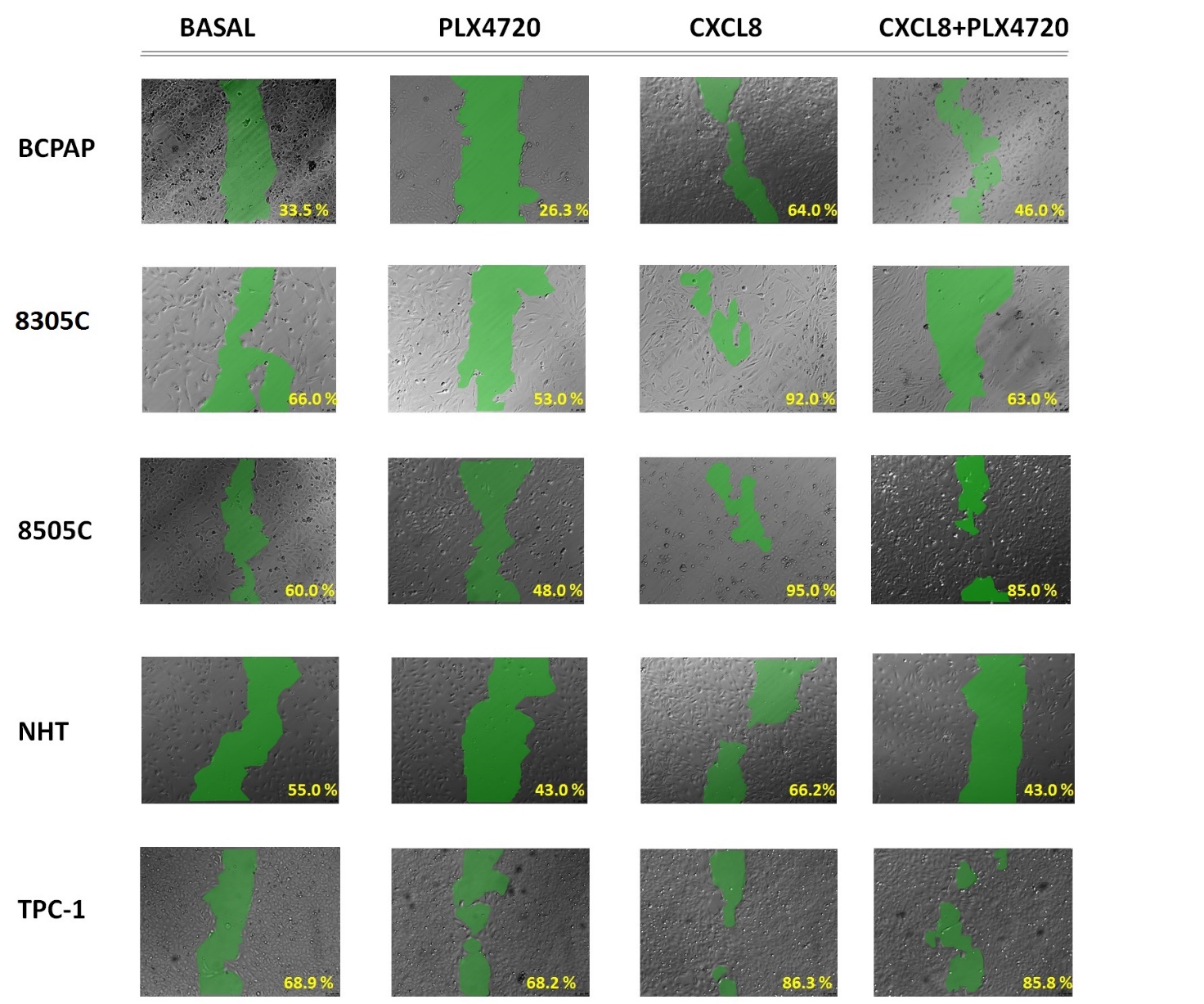


**Figure S2: Representative images of Wound healing assay after 24hrs for BCPAP, 8305C, 8505C, NHT and TPC-1. BCPAP:** the addition of PLX4720 10µM reduced the wound closure from 33.5% (of the basal migration) to 26.3% (after treatment with PLX4720) in BCPAP, incubation with rh-CXCL8 increased wound closure to 64% which was reduced by co-treatment with PLX4720 to 46% (rh-CXCL8+PLX4720). **8305C:** the addition of PLX4720 10µM reduced the wound closure from 66% (of the basal migration) to 53% (after treatment with PLX4720), incubation with rh-CXCL8 increased wound closure to 92%, which was reduced by co-treatment with PLX4720 10µM to 63% (rh-CXCL8+PLX4720). **8505C:** the addition of PLX4720 10µM reduced the wound closure from 60% (of the basal migration) to 48% (after treatment with PLX4720), incubation with rh-CXCL8 increased wound closure to 95.5% which was reduced by co-treatment with PLX4720 10µM to 85%. **NHT:** the addition of PLX4720 10µM reduced the wound closure from 55% (of the basal migration) to 43% (after treatment with PLX4720), incubation with rh-CXCL8 increased wound closure to 66.2% which was reduced by co-treatment with PLX4720 10µM to 43% (rh-CXCL8+PLX4720). **TPC-1:** no inhibitory effect was observed after the addition of PLX4720 on cell migration from 68.9% of the basal migration (to 68.2%), incubation with rh-CXCL8 increased wound closure to 86.3% but the co-incubation with PLX4720 did not reduce it (85.8%).
